# A Preventive Role of Exercise Across the Coronavirus 2 (SARS-CoV-2) Pandemic

**DOI:** 10.3389/fphys.2020.572718

**Published:** 2020-09-08

**Authors:** Meizi Wang, Julien S. Baker, Wenjing Quan, Siqin Shen, Gusztáv Fekete, Yaodong Gu

**Affiliations:** ^1^Faculty of Sports Science, Ningbo University, Ningbo, China; ^2^Faculty of Engineering, University of Pannonia, Veszprém, Hungary; ^3^Department of Sport, Physical Education and Health, Centre for Health and Exercise Science Research, Hong Kong Baptist University, Kowloon Tong, Hong Kong; ^4^Savaria Institute of Technology, Eötvös Loránd University, Szombathely, Hungary

**Keywords:** coronavirus 2019, obesity, overweight, exercise, health

## Abstract

The coronavirus 2019 (COVID-19) pandemic has posed a significant threat to human health around the world. A severe risk of infection has been observed in elderly populations. In addition, individuals with obesity and obesity-related comorbidities have also been identified to be at a higher risk of infection for COVID-19. We have attempted here to provide evidence in support of exercise management as a prevention strategy for improving health and minimizing the effects of COVID-19. Therefore, exercise duration, frequency, and intensity benefits are summarized in an attempt to provide guidelines for the general population. In terms of exercise effects, there are multiple benefits of exercise related to human health. These include, decreases in adipose tissue, improvements in cardio-respiratory fitness, enhanced metabolic homeostasis, and suppress inflammation active. With respect to the amount of exercise performed individuals should exercise at a moderate intensity for at least 150 min/wk as an initial target. Increases in intensity and duration of exercise training are necessary for significant fitness benefits, weight loss, and prevention of weight regain. In relation to walking, 10,000 steps/day at a rate of 64–170 steps/minute for at least 10 min duration is reasonable for healthy adults. For exercise intensity, a combination of resistance training (RT), aerobic training (AT) as well as high-intensity interval training (HIIT) incorporated with moderate-intensity continuous training (MICT) can be recognized as an optimal exercise mode for health benefits. Aerobic training and MICT should be viewed as a basis for exercise in combination with appropriate volumes and types of RT and HIIT. Activities should be performed according to professional guidelines and advice. If implemented, these measures may reduce infection rates, underlying pathologies, and assist in decreasing mortality associated with COVID-19 pandemic.

## Introduction

The coronavirus disease 2019 (COVID-19) has recently become one of the greatest threats the world has faced. 6,976045 cases of severe acute respiratory syndrome coronavirus 2 (SARS-CoV-2) infection has been confirmed in more than 100 countries based on the latest World Health Organization (WHO) report (07 June 2020) (World Health Organization [WHO], 2020). Among these cases, it appears that new pandemic complications are already well-defined in elderly populations. These include (age ≥ 60 years), obese and overweight people with body mass indexes (BMI) over 25 kg/m^2^ or even higher contribute to increased risk infection scores for SARS-CoV-2. According to a report published by the Intensive Care National Audit and Research Centre (ICNARC) they have been informed of 11,634 admissions for critical care with confirmed SARS-CoV-2 (22th May 2020). It was reported that 73.41% of patients confirmed with SARS-CoV-2 were overweight or obese (ICNARC, 2020). In addition, Italian data published on 21st May 2020 indicated that the overall prevalence of obesity was 11% among 3032 dying cases (Epicentro, 2020). Recently, a report from the United States in New York City found that, among patients aged <60 years with SARS-CoV-2 who were positive symptomatic, patients who were obese (30 < BMI < 34) were 1.8–2.0 times more likely to receive critical care, than those with a BMI < 30 (Chiappetta et al., 2020). The effects of BMI are further substantiated by preliminary data from China, among 383 patients with SARS-CoV-2 in Shenzhen city, overweight was associated with 86%, and obesity was associated with 42% of patients (Cai et al., 2020).

**FIGURE 1:**
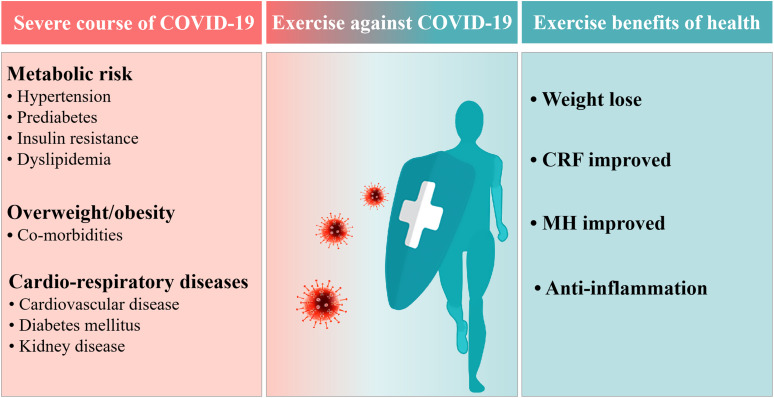
Exercise benefits against coronavirus disease in 2019 (COVID-19). CRF, Cardio-respiratory fitness; MH, Metabolic homeostasis.

Obesity is defined as a condition of excess body fat and represents a state of low-grade chronic inflammation and impaired immunity that is associated with many debilitating and life-threatening disorders. These include respiratory dysfunction, cardiovascular disease, diabetes, some cancers, metabolic risk, and associated co-morbidities, some of which are widely recognized as being related with more severe COVID-19 (Stefan et al., 2020) (Figure 1). Although the exact mechanism by which obesity contributes to severe COVID-19 has not been determined, several theories may provide an explanation. From a lung function perspective, patients with excess adipose tissue could promote the existence of ectopic adipocytes in the alveolar interstitial space which may be directly exposed to viral infection, exacerbating inflammatory infiltrations and leading to substantial interstitial edema (Watanabe et al., 2020). With respect to physiochemical reactions, the Angiotensin-converting enzyme 2(ACE2) has been identified as a receptor for COVID-19 entry and obesity could facilitate a higher expression of ACE2 in lung epithelial cells. This highlights that the more adipocytes present, the more ACE2 receptor concentrations to spread the virus (Jia et al., 2020). There is also an ineligible association between infection state and expression of ACE2 (Jia et al., 2005; Al Heialy et al., 2020).

Overweight and obesity are increasing at an alarming rate globally and the problem has reached epidemic proportions in almost every country. Is this the time to promote and reinvigorate the exercise for health agenda? Could the COVID-19 effects have been minimized between genders, ages and ethnicities if the World was healthier and participating in regular physical activity? Are governments and policymakers now urgently required to promote physical activity practice for all ages and ethnicities? In the early stages of childhood and adolescence, weight gain progression to morbid obesity can be changed by exercise and diet modifications. This is possible without the need for medication, endoscopic or surgical procedures. We will outline here evidence in support of the current best practices in exercise management as a prevention strategy that could be useful in the management and minimization of COVID-19.

## Exercise Benefits for Health and COVID-19

The positive impacts of exercising on human health have been attributed to several mechanisms, which involve decreased adipose tissue, increased cardio-respiratory fitness determined by maximal oxygen consumption (V02 max) and metabolic homeostasis improvements. The following statements will be developed further defining and outlining these mechanisms.

Increasing daily energy expenditure through exercise training or physical activity is an effective way to lose weight. Specifically, exercise accelerates the breakdown of glycogen in the muscles and liver; this promotes the breakdown of fat in adipose tissue and muscle; and facilitates the oxidation of fatty acids in muscles (Poirier and Després, 2001). All these effects are mediated through hormone stimulation, secretion and substrate concentration, leading to enzyme activation, which is a key step in the catalytic energy catabolism pathway (Moinuddin et al., 2012). Typically, according to a previous study demonstrating that exercising about 60 min at moderate training more than 4 days a week, individuals will lose 0.5 lb per week (Poirier and Després, 2001). A review presented the positive impacts of aerobic exercise on obese people, which revealed a significant reduction in weight, waist circumference and fat mass (Schwingshackl et al., 2013). Although, exercising is a primary way to fight obesity, combining a strategy of reducing energy intake rather than solely exercise intervention is also important (Goodpaster et al., 2010; Slentz et al., 2011; Martin et al., 2019).

Cardio-respiratory fitness (CRF) is inversely related to visceral adipose tissue accumulation (overweight and obesity) (Brock et al., 2011). Mounting evidence has established a strong relationship between lower CRF and a high risk of cardiovascular disease (CVD), morbidity, and mortality attributable to various cancers (Lee et al., 2010; Ross et al., 2016). Previous research has indicated that physical activity is the primary modifiable determinant of CRF, where both critical factors of central and peripheral adaptations to CRF can be significantly improved by exercising (Saltin et al., 1976; Weiner and Baggish, 2012). Oxygen delivery and oxygen uptake in the exercising muscles are enhanced as a result of exercise training, ultimately improving CRF. The CRF response to exercise training in female subjects aged from 45 to 75 years has been previously investigated (Church et al., 2007). Statistically significant increases in peak absolute oxygen consumption (V02abs, L/min) by 4.2, 6.0, 8.2% in the 4, 8, and 12 kcal/kg energy-expenditure groups were noted, respectively, per week over a period of 6 months when average baseline of V02abs was 1.30 (0.25) L/min (Ho et al., 2013). Relevant studies in subjects with low baseline CRF have demonstrated significant improvements in CRF using exercise interventions of different modalities (aerobic and resistance) at moderate intensities in middle-aged overweight women and men (Church et al., 2010). Ross et al. reported effective results that exercise performed for 30 min per day a week increased CRF by 9.4%, exercise performed for 60 min per day at the same intensity and duration increased CRF by 15.6% (Ross et al., 2015). Further details are available in the scientific statement from the American Heart Association (Ross et al., 2016). These results show a dose response effect for CRF improvement for both intensity and volume of physical activity in initially inactive overweight men and women.

The disruption of metabolic homeostasis is associated with physical inactivity, characterized by insulin sensitivity, post-prandial lipid clearance, decreased muscle mass and increased visceral adiposity (Pedersen and Febbraio, 2012). The metabolic syndrome involving hypertension, insulin resistance, dyslipidemia and diabetes describes common pathological and physiological characteristics (Eckel et al., 2005). This syndrome was found in a group of both men and women who had 4.6% normal-weight, 22.4% overweight and 59% obesity, respectively (Golbidi et al., 2012). In addition, Nunn et al. (2010) indicated that chronic systemic inflammation related to metabolic disruption could be a key factor related to persistent physical inactivity. Therefore, increased exercise training is likely to be an effective way to prevent metabolic derangement. Guidelines support that the prevalence of metabolic syndrome can be lowered by performing the moderate-intensity exercise for at least 150 min per week (Donnelly et al., 2009). The lowest prevalence of metabolic syndrome is observed in populations involved in high intensity and continuous exercise of more than 2 h per week. Low-volume activity may not present such a significant influence (Hahn et al., 2009).

Additionally, the previously published data from a large population cohort researches consistently noted a negative relationship between inflammation and exercise training. COVID-19 is characterized by an overactive inflammatory response, the inflammation cytokines like IL-6, IL-1β, and TNF-alpha were overproduced as a necessary response of the immune system to infection when a virus attacks the immune cells, damage to lung parenchyma and bronchi, causing adverse respiratory disturbances, and further severe enough to require medication (Jose and Manuel, 2020; Stebbing et al., 2020). However, the lower inflammatory biomarker (IL-6, IL-1β, and TNF-alpha) are found in individuals who conducting more frequent and intense physical activity (Gleeson et al., 2006; Beavers et al., 2010). Above all, exercise provides multiple benefits to boost individual’s health, which may also be beneficial to potentially reduce the risk of infections; this is currently relevant, considering the situation of COVID-19 pandemic which is still ongoing with the absence of a vaccine.

## Appropriate Duration and Frequency of Exercise

The physical activity guidelines are based on duration, frequency, and intensity of exercise acknowledged by the public for providing information about: “How much physical activity is enough” and “how much exercise is needed to benefit health.” In 2017, American College of Sports Medicine(ACSM) exercise testing and prescription presented a weight management goal for adults which based on the previously published data in 2007, the suggestions are summarized below: (1) 150–250 min/wk^–1^ (approximately 1200–2000 kcal/wk^–1^) of moderate-intensity physical activity is sufficient to prevent initial weight gain; (2) more than 250 min/wk^–1^ is related to clinically significant weight loss; (3) 250–300 min/wk^–1^ (approximately 2000 kcal/wk^–1^) to maintain weight loss (American College of Sports Medicine [ACSM], 2017). Furthermore, in 2018, the ACSM (a complete guide to fitness and health) emphasized the importance of a target of 150 min/wk^–1^ moderate-intensity activity for health (Bushman and American College of Sports Medicine [ACSM], 2017). Additionally, the World Health Organization (WHO) recently released a global plan on physical activity for 2018 to 2030 also recommend that it is an effective way to enhance health for adults by performing moderate to vigorous physical activity in 30–60 min at least 5 days per week (World Health Organization [WHO], 2019). Therefore, 150 min/wk exercise duration is a basic amount for keeping health in the normal population. But for weight loss, the amount of weight reduction was significantly associated with the duration of exercise conducted. To be more specific, another research indicated that have moderate-intensity physical exercise everyday instead of only 3–4 times/wk contributed to a clinically significant weight loss (Swift et al., 2018). Similarly, a systematic review by Damon et al. summarized that the incidence of clinically significant weight loss is low (<20%) among individuals who do not exercise every day and exercise <2000 kcal/week (Donnelly et al., 2013). Furthermore, in a 24 wk weight loss program, Madjd et al. (2016) reported results of a randomized controlled trial of 75 obese women that a longer duration exercise was more effective for weight decrease compared to a more frequency shorter session.

For anaerobic training duration and frequency, the RT or HIIT refers to a repeated session of brief intermittent training which can be conducted by a single effort lasting a few second to several minutes (Xie et al., 2017), a typical HIIT session involves 4 × 4 min interval with 3 min recovery as well as 4 × 5 min (3 min recovery), 3 × 5 min (3 min recovery), 10 × 30 s (1 min recovery), etc. (Karlsen et al., 2017). On the other hand, the RT and HIIT are not a proper method as the primary plan of obesity reduction if participants aim to lose weight, and it should be combined with aerobic training, more details for this are discussed below. It is also important to note that the intensity and volume of RT and HIIT are related to an individual’s VO_2__peak_ and HR_peak_, each participant should be tailored individually with professional guidelines. Therefore, the suggestion of exercise duration and frequency of RT and HIIT are limited here. In the summary, individuals should perform the exercise at a moderate intensity for at least 150 min/wk with more than 30 min each time for aerobic exercise corporation with the appropriate RT and HIIT as an initial target. Following the initial phase, a longer duration exercise is necessary for significant weight loss and prevention of weight regain.

In addition, one point needs to be highlighted here that is related to the amount of walking exercise. Walking is the most popular, affordable and readily available form of moderate-intensity physical activity which attracts a lot of people from different ages around the world. Walking steps can be accumulated during everyday activities such as transportation, shopping, occupational requirements and chores. Walking is also easily monitored by portable pedometers and mobile applications to provide timely feedback for users. Regarding the topic of “how many steps/day are enough? for adults” has been summarized by Tudor-Locke et al. (2011). The recommendations are presented as: (1) 10,000 steps/day is reasonable for healthy adults; (2) 64–170 steps/minute with at least 10 min duration is also reasonable for healthy adults. Although the steps for children and adolescents have been objectively monitored (Tudor-Locke et al., 2011), there is still a lack of specific guidelines to determine how many steps are reasonable for healthy growth and development.

## Exercise Intensity

### Resistance and Aerobic Exercise Training

Resistance training is a type of exercise that causes the muscles to contract against an external resistance with the purpose of improved muscle strength, size and endurance. RT enhances muscular strength, which provides free-living physical activity, by increasing energy expenditure to improve metabolic rate (Donnelly et al., 2009). However, the vast majority of data suggest that RT only leads to a minimal decrease in body fat (US Department of Health and Human Services, 2008; Donnelly et al., 2009; Swift et al., 2014). Some studies have observed only moderate weight loss after 16 to 26 weeks of continuous RT performed (Lemmer et al., 2001; Hu et al., 2003; Ibañez et al., 2005). Another study found no effects on weight loss after a 12–52 weeks continuous RT intervention (Lemmer et al., 2001; Polak et al., 2005; Ferrara et al., 2006; Olson et al., 2007). Although the effects of RT on weight loss may be slight, RT can significantly improve CVD risk factors without significant weight loss (Donnelly et al., 2009). Also, reductions in fat may be accompanied by increased lean tissue mass. This will result in little change in overall body mass profiles, but will positively improve body composition parameters. According to previous research reductions in HDL-C, LDL-C, and TG, enhanced insulin sensitivity and decreases in glucose-stimulated plasma insulin concentrations have been found following RT (Ibañez et al., 2005; DiPietro et al., 2006). In addition, systolic and diastolic blood pressure was reduced following RT (Patel et al., 2017). RT may not seem to be an effective way to reduce body fat, but it is connected with many other positive influences including decreased risk of chronic diseases and increases in lean muscle mass.

According to the ACSM definition of aerobic training (AT) the large muscle groups are the ones most responsive and need to be continuously stimulated while providing natural rhythm in any activity (O’Sullivan et al., 2019). It is a traditional way to reduce body fat, improve the capacity of CRF to supply oxygen, and enhance the ability to skeletal muscles to use oxygen (Booth and Winder, 2005). Some cross-sectional and longitudinal intervention studies have confirmed that relative risk factors associated with metabolic diseases could be reduced by prolonged AT such as continuous running, walking, swimming or cycling (Hawley, 2004; Booth and Winder, 2005; Pedersen and Saltin, 2006).

The exercise mode of RT and AT has been widely discussed. Cris et al. investigated the effects of three different exercise modalities (RT, AT, RT combined with AT) on 249 subjects’ body composition. The results found that AT was more effective in decreasing visceral and liver fat and total abdominal fat compared to RT. Resistance training led to a reduction in subcutaneous abdominal fat, and the effect of RT combined with AT was not statistically different from AT (Slentz et al., 2011). Additionally, another study observed that a group of AT and AT/RT had a significant decrease in total fat mass more than RT alone, while RT and AT/RT resulted in a significant increase in lean body mass than AT alone (Willis et al., 2012). Although both these modes of exercise (RT and AT) play a positive role in body weight control and health, these variations were caused by different mechanisms. This has resulted in RT stimulating a larger lean body mass and AT reducing total fat mass (Willis et al., 2012). In summary both RT and AT provide a wide variety of health benefits for humans. RT promotes improvements in lean body mass, strength and muscle mass, and AT is superior in managing total body weight. According to the updated version of the 2007 American Heart Association Council, it states that RT should be incorporated with AT to improve body health and weight (Williams et al., 2007). Research investigating 136 abdominally obese men and women with older ages who exercised for 6 months found that performing a combination of RT and AT was the optimal exercise combination to improve cardiopulmonary fitness and insulin sensitivity. In addition, larger decreases were observed in abdominal and visceral fat by performing RT and AT, respectively (Davidson et al., 2009). Therefore, a combination of RT and AT can be recognized as an optimal exercise mode for health benefits.

### High Intensity Interval Training and Moderate-Intensity Continues Training

High-intensity interval training is defined as exercise at maximum intensity using peak oxygen uptake consumption (≥90% of V02 max) for a short period followed by periods of rest (30–60 s). This method has been widely utilized by athletes to optimize movement and sports performance. The method has also gained in popularity as an intervention strategy for weight management in the general population (Obert et al., 2017). HIIT is greatly different from MICT which is characterized by 30–60% of V02 max.

Many publications are comparing the effects of HIIT and MICT on body composition. According to the American National Health and Nutrition (ANHN) examination survey, they demonstrated that high HIIT contributes to a lower BMI, and that moderate to vigorous-intensity exercise is superior for weight reduction than MICT alone (Fan et al., 2013). A meta-analysis by Türk et al. (2017) observed a significant decrease in body fat after a HIIT intervention in contrast to MICT. Racil et al. (2013) have investigated 34 obese adolescent women performing HIIT and MICT, respectively, for 12 weeks. The results showed that body mass and waist circumference decreases were significantly greater in HIIT compared to MICT. There was also an inconsistent opinion that a meta-analysis including 13 previous studies showed a moderate effect for decreasing body fat between HIIT and MICT (Wewege et al., 2017).

Despite the conflicting observations on the effects of HIIT and MICT on body fat, there is confirmation that HIIT plays a more effective role in enhancing V02max, metabolic homeostasis, vascular function and the immune system than MICT. Based on several published studies HIIT resulted in a significant increase in V02max and muscle oxidative capacity compared to MICT. HIIT was more optimal in decreasing risk factors that cause metabolic syndrome than MICT (Swisher, 2010; Czernichow et al., 2011; Vakil et al., 2012). Additionally, Petridou et al. also showed a better therapeutic influence on cardiovascular and health-related quality of life outcomes by performing HIIT for 12 weeks than MICT (Petridou et al., 2019). According to a recently published article, the suggestion was that only MICT (not HIIT) should be recommended as an effective exercise mode to cope with COVID-19 because HIIT leads to suppression of the immune system and induces an inflammatory response (Rahmati-Ahmadabad and Hosseini, 2020). Actually, Campbell and Turner (2018), indicated that there was strong evidence demonstrating that a decrease in the function of lymphocytes in peripheral blood and other immune cells after HIIT does not reflect immunosuppression. Instead, this reduction represents an enhanced state of immune surveillance and regulation. Furthermore, according to relevant articles observed that HIIT rather than performing a long intense exercise can decrease the pro-inflammatory and increase the anti-inflammatory indices (Munk et al., 2011; Steckling et al., 2016). Therefore, it seems that HIIT may be an acceptable as part of a regular exercise regime in daily life which could help to cope with COVID-19. However, it should be noted that HIIT is associated with a higher risk of musculoskeletal injury, and needs to be performed under professional guidance or advice. In summary, a combination of HIIT and MICT exercise is a reasonable way to gain health benefits, manage body fat, and consider as a potential way to minimize the effects of COVID-19 infection. Specifically, MICT should be a foundation of exercise in combination with the appropriate type and volume of HIIT for daily exercise.

Exercise benefits, duration, frequency, and intensity are summarized here tried to provide a suggestion for the general population, which include the overweight/obese and the unfit. The advice could provide stimulation for the human constitution and could play a potential effect on resisting the virus. In terms of exercise benefits, there are multiple advantages of exercise in relation to human health. These include decreased adipose tissue, improved cardio-respiratory fitness, enhancement of metabolic homeostasis and even suppress inflammation active. Additionally, with respect to the necessary amount of exercise, individuals should perform the exercise at a moderate intensity of at least 150 min/wk initially. This should be followed by a larger amount of exercise for significant weight loss and to prevent weight regain. As for the amount of walking, 10,000 steps/day in which 64–170 steps/minute with at least 10 min duration is reasonable for healthy adults. In relation to exercise intensity, a combination of RT and AT as well as MICT incorporated with HIIT can be recognized as an optimal exercise mode to benefit health. AT and MICT should be viewed as a basis for exercise, then a combined appropriate volume and intensity variation of RT and HIIT should comply with professional guidelines (Table 1). These measures if implemented sooner may have reduced underlying pathologies and contributed to the reduction of mortality associated with COVID-19 pandemic. Future generations, governments, and policymakers need to be aware of the health benefits of exercise and its potential for saving lives. In addition, some extra tips must be mentioned in here that it is important to keep social distancing and avoid mass gatherings in public area while performing exercise under the condition of COVID-19 still ongoing around the world, and developing in-house exercise schedules consisting of different exercise types is also recommended. Furthermore, if there any symptom of some diseases occur, exercise should be reduced or avoided according to the doctor’s instructions.

**TABLE 1:** The general suggestion for improving public health to against COVID-19.

	**Aerobic exercise (MICT)**		**Anaerobic exercise (RT and HIIT)**	
	**Exercise duration**	**Exercise frequency**	**Exercise duration**	**Exercise frequency**
A initial target for normal population	≥150 min/wk; ≥30 min per time	3–5days/wk	4 × 4 min (3 min recovery); 4 × 5 min (3 min irecovery); 3 × 5 min (3 min recovery); 10 × 30 s (1 min recovery); etc.	Should to be tailored individually with professional characteristics
	10,000 steps/day; 64–170 steps/min with at least 10 min duration	Everyday	–	–
For weight loss purpose	>250 min/wk; >30 min per time	>5 days/wk	4 × 4 min (3 min recovery); 4 × 5 min (3 min recovery); 3 × 5 min (3 min recovery); 10 × 30 s (1 min recovery); etc.	Should to be tailored individually with professional characteristics

*RT, resistance training; MICT, moderate-intensity continuous training; HIIT, high intensity interval training.*

## Author Contributions

JB, GF, and YG developed the study concept. MW, SS, and WQ contributed to the intellectual content and editing of the manuscript. JB and YG approved the final version. All authors contributed to the article and approved the submitted version.

## Conflict of Interest

The authors declare that the research was conducted in the absence of any commercial or financial relationships that could be construed as a potential conflict of interest.
